# Supplementary material for: Genome-wide identification and characterization of the KCS gene family in sorghum (Sorghum bicolor (L.) Moench)
Source: PeerJ. 2022 Oct 7;10:e14156. doi: 10.7717/peerj.14156 (PMC9549899; doi:10.7717/peerj.14156)
Supplement: Figure S1 [file peerj-10-14156-s005.pdf]

```
                   *        20         *        40         *        60         *        80
AtKCS1    IEMDRERLTAEMAFRDSS------SAVIRIRRRLPDLLTSVKLKYVKLGLHNSCNVTLFFLIILPLTGTVLVQLTGLTFD :  74
AtKCS2    --MNENHIQSDHMNN---TIHVTN-------KKLPNFLLSVRLKYVKLGYHYLISNAILILP-VGLLAAT-SSSFSLTD- :  65
AtKCS3    ------------------------------------------------------------MDLLVMLLSLLVS------- :  13
AtKCS4    -----------MDGAGESGGDGSVGVQIRQTRMLPDFLQSVNLKYVKLGYHYLISNLLTLCLFPLAVVISVEASQMNPDD :  69
AtKCS5    ---------------------------------MSDFSSSVKLKYVKLGYQYLINNFLTLLLIPVIATVAIELLRMGPEE :  47
AtKCS6    ----------------------------MPQAPMPEFSSSVKLKYVKLGYQYLVNHFLSFLLIPIMAIVAVELLRMGPEE :  52
AtKCS7    --------------------------------------MESSFHFINE------------ALLITQTFITFHQFLVAS-- :  28
AtKCS8    ------------------------------------------MKNLKMVF-------FKILFISLMAGLAMKGSKINVED :  31
AtKCS9    -----------MEAANEPGGSVQIRTENNERRKLPNFLQSVNMKYVKLGYHYLITHLFKLCLVPLMAVLVTEISRLTTDD :  69
AtKCS10   -MGRSNEQDLLSTEIVNRPNAGSPTFSVRVRRRLPDFLQSVNLKYVKLGYHYLINHAYLATIPVLVLVFSAEVGSLSREE :  79
AtKCS11   --MDVEQKKP--------LIESSD-------RNLPDFKKSVKLKYVKLGYHYLITHGLFLSPLVLVIAAQ-ISTFSVTD- :  61
AtKCS12   ------------------------------------------------------------MDLLFLFFSLLLS------- :  13
AtKCS13   -----------------------------------MFIAMADFKILLL-----------ILILISLFELDLLHFHHDFFS :  34
AtKCS14   -----------------------------------MFIAMADFKLLLL-----------ILILLSLFELDLLHFHHDFFS :  34
AtKCS15   -------MEKEATKMVNGP-KGS-----------PDFLG-YNLRYVKLGYIYLLSLSFCFFLPPLLLFIFVSRFLP--- :  57
AtKCS16   --------------------------------------MDYPMKKVKIFFNYLMAHRFKLCFLPLMVAIAVEASRLSTQD :  42
AtKCS17   -----------MDAN---GGPVQIRTQN----------------YVKLGYHYLITHFFKLMFLPLMAVLFMNVSLLSLN- :  49
AtKCS18   ----------------------------------------MTSVNVKLLYRYVLTNFFNLCLFPLTAFLAGKASRLTIND :  40
AtKCS19   ------------------------------------------------------------MELFSLSSLLLLST------ :  14
AtKCS20   --MSHNQNQ-PHRPV---PVHVTNAEPNPNPNNLPNFLLSVRLKYVKLGYHYLISNAILLLPLLAATIAN-LSSFTIND- :  72
AtKCS21   --------------------------------------MNQTIHRVSP------------ISMSISELTTLLSSGVSVF- :  29
SbKCS1    ------------------------------------------------------------MELMTLLTLPLLAC------ :  14
SbKCS2    -------------------------------------------------------------------------------- :   -
SbKCS3    AVMERERLTAELDLAVV-AAEPS-SFVVKIRRRLPDFARSVNLKYVRLGLRSGGIPATWVPLALAPP---LLAAAVYSLV :  75
SbKCS4    ------------------------------------------------------------MELLPLLTWLLLAH------ :  14
SbKCS5    AVMERERLTAEMAFRGADGGEPAPSIVIKIRRRLPDFARNIKLKYVKLGIRHGGSPT--VLPMLCVP---AIAAAAYSFV :  75
SbKCS6    ----------------------------MPTG--GVFSGSVNLKYVKLGYQYLVNHFLTLLLVPVMAATALELARLGPGE :  50
SbKCS7    -MAR-EEQALLSTEIMNRPDAGSPTFSVRVRRRLPDFLQSVNLKYVRLGYHYLISHGYLATIPVIVLVCGAEVGSLSRDE :  78
SbKCS8    --MANQQHRSSIAGMD---------------RELFRTVRQAALNHARLLYHRLVSRLLLAVTLVVAAAQLVPPPSSAHA- :  62
SbKCS9    ---------------------------------------------MLSSKAAAAAAAASLRTLPLPVLVPLVVS------ :  29
SbKCS10   -------------------------------------MDIAHRDYLQVTLHTILGVALLCVLAELFIFAFRCRTALYLVP :  43
SbKCS11   ---------------------------------MHTRKTMAKLLSLKTWYQLFVDNFIVAVAFVAAALRRAWPVS-VDDL :  46
SbKCS12   ------------------------------------MNTPLHVKFLKTMYRLVVNNFVVALAVAAAVVPRTSPH---EEV :  41
SbKCS13   --MENPAPPTDPAAATGAPSSSPS-------RQLPDFQQSVRLKYVKLGYHYLISHGLLLTPLMVLVAVH-LSTLSPRD- :  69
SbKCS14   --MSAAHQPEPTAPA----AAASCGGGGDSTIKNARHRT-STLKQCYH---LAISNALLLAP-AVAYASHRFSHHTASD- :  68
SbKCS15   --MSIKSETNERS----------------STIQSAGHRICSIVKLCYQHLMLAVSYALFLAPIILATLVLHLSRHTSADD :  62
SbKCS16   -------------------------------------------------------------------------------- :   -
SbKCS17   -----------------------------------------------MLSHLGMS-----VAAAAAMLQRAWTFS-YGII :  27
SbKCS18   -----------MNGGAAPAASAAAAAATPPHRRLPDFLQSVNLKYVKLGYHYLITHLLTLMLLPLMAVILLEAGRTDPDD :  69
SbKCS19   --MADEPRTT--------AAATPS-------SRMPDFKQSVKLKYVKLGYHHLISHGLLLAPLPGLVAAH-LSTFTLRD- :  61
SbKCS20   ---------------------------------MSSPPLFGNGKGLKAVCRRVVDNIVVTVPLTAAALVAMARFGPEEQL :  47
SbKCS21   ---------------------------------MALS---PSQRLLRSLYQIVVNNVTIAVSLTTAVILVAAQFG-FSEH :  43
SbKCS22   ---------------------------------MSSS---HIGKYLKTVFAKIVDNFTVAVPFATAAVIVAATTSRSDEF :  44
SbKCS23   ---------------------------------MNSL---LSLKRLKYVFQLVVNNFTVAVLFAAILLRKGAQLG-LAEI :  43
SbKCS24   --------------------------------------------------------------MIAMILEVTQQLCQDRLL :  18
SbKCS25   --METSAPPNAAAP----PAQQPR-------RRLPDFQQSVRLKYVKLGYHYLISHGLLLSPLMALVAVQ-LSTVSPRD- :  65

                   *       100         *       120         *       140         *       160
AtKCS1    TFSELWSVQLDTATRLTCLVFLSFVLTLYVANRSKPVYLVDFSCYKPEDERKISVDSFLTMTEENGSFTDDTVQFQQRIS : 154
AtKCS2    -LTLLYNLKFHFLSSTLFAALLIFLTTLYFTTRPRRIFLLDFACYKPDSSLICTRETFMDRSQRVGIFTEDNLAFQQKIL : 144
AtKCS3    ------------------YLIFKIWKRIDSKRDQ-NCYILDYQCHKPSDDRMVNTQFSGDIILRNKHLRLNE-KFLLKAI :  73
AtKCS4    LKQLWIHLQYNLVSIIICSAILVFGLTVYVMTRPRPVYLVDFSCYLPPDHLKAPYARFMEHSRLTGDFDDSALEFQRKIL : 149
AtKCS5    ILSVLNSLHFELLHILCSSFLIIFVSTVYFMSKPRTVYLVDYSCYKPPVTCRVPFSSFMEHSRLILKDNPKSVEFQMRIL : 127
AtKCS6    ILNVWNSLQFDLVQVLCSSFFVIFISTVYFMSKPRTIYLVDYSCYKPPVTCRVPFATFMEHSRLILKDKPKSVEFQMRIL : 132
AtKCS7    ------------------ACVLIAVFGYYFFKPRCIIYLIDFSCYQPPDFLRAPVSNFIEHLTISGVFDQESLDLQQKIL :  90
AtKCS8    LQKFSLHTQNNLQTISLLLFLVVFVWILYMLTRPKPVYLVDFSCYLPPSHLKVSIQTLMGHARRAREAGMCWVDFQEKIL : 111
AtKCS9    LYQIWLHLQYNLVAFIFLSALAIFGSTVYIMSRPRSVYLVDYSCYLPPESLQVKYQKFMDHSKLIEDFNESSLEFQRKIL : 149
AtKCS10   IWKKLW--DYDLATVIGFFGVFVLTACVYFMSRPRSVYLIDFACYKPSDEHKVTKEEFIELARKSGKFDEETLGFKKRIL : 157
AtKCS11   -LRSLWELQYNLISVVVCSMLLVFLMTIYFMTRPRPVYLVNFSCFKPDESRKCTKKIFMDRSKLTGSFTEENLEFQRKIL : 140
AtKCS12   ------------------YLFFKIWKLIDSKQDK-DCYILDYQCHKPTDDRMVSTQFSGEIIYRNQNLGLTE-KFLLKAI :  73
AtKCS13   PFPVKIG-----------LLLISIFFYAYSTTRSKPVYLVDFSCHQPTDSCKISSETFFNMAKGAQLYTDETIQFMTRIL : 103
AtKCS14   PFPVKIG-----------LLLISIFFYAYSTTRSKPVYLVDFSCHQPTDSCKISSETFFNMAKGAQLYTEETIQFMTRIL : 103
AtKCS15   --------------ILAFPLSTFFILLIYHYLTPSSVFLLDFSCYRPPDHLKITKSDFIELAMKSGNFNETAIELQRKVL : 123
AtKCS16   LQNFYLYLQNNHTSLTMFFLYLALGSTIYLMTRPKPVYLVDFSCYLPPSHLKASTQRIMQHVRLVREAG-AWMDFCEKIL : 121
AtKCS17   ----HLQLYYNSTGFIFVITLAIVGSIVFFMSRPRSIYLLDYSCYLPPSSQKVSYQKFMNNSSLIQDFSETSLEFQRKIL : 125
AtKCS18   LHNFLSYLQHNLITVTLLFAFTVFGLVIYIVTRPNPVYLVDYSCYLPPPHLKVSVSKVMDIFYQIRKADTSSLDFLRKIQ : 120
AtKCS19   ------------------LFVFYIFKFVFKRRNQRNCYMLHYECYKGMEERKLDTETCAKVVQRNKNLGLEE-RFLLRTM :  75
AtKCS20   -LSLLYNLRFHFLSATLATALLISLSTAYFTTRPRRVFLLDFSCYKPDPSLICTRETFMDRSQRVGIFTEDNLAFQQKIL : 151
AtKCS21   ---------------EIFAGLLVVHLIYQRIRTRVKVYLLDFTCYRAPDSNRVPMSTLIETIYLDDKLDQESIDFQARIL :  94
SbKCS1    ------------------LAAMLARLLVARAQRR-RCYLLDYVCYKATDDRKLPTDLCGEIIQRNQLLGLEE-KFLLKVI :  74
SbKCS2    ---KEMEVGLSPAAVAVACWAAAAAAWAYAVSRPRPVYLVDLSGYVAGAAHEASRAKTIAHFGRCGRFSDESMAFQKRML :  77
SbKCS3    GADKLY--SLDLLTCVAWLAAAVLLLTVYFLKRPRPVYLVDFACYRPADEHAISKEGFLDMTESTGWFNAEALEFQTKIT : 153
SbKCS4    ------------------AMAYLAWTAAARRRQS-RCYLLDYVCHKPRDDRKLSTETAGDVIQRNARLGLTD-RFLLRVV :  74
SbKCS5    RLDVIYY-SIDLLTCVAWLGTALLLLTVYYFKRPRPVYLVEFACYKPEEQLKISKSAFLEMTESTGSFNEAALDFQTKIT : 154
SbKCS6    LLSLWRSLELDLVHILCSVFLVVFIGTVYVMSRPRPVYLVDYACYKPPASCRVPFATFMEHTRLISDDD-KSVRFQTRIL : 129
SbKCS7    LWRKVWGATYDLATVLAFLAVLAFTISVYIMSRPRPVYLIDFACYKPADELKVSKAEFIDLARKSGKFDEDSLAFQSRLL : 158
SbKCS8    -ARTLLRAARADAGLVAAALAACACACAYAALRPRPVYLVDLAAYRPGPAHRATRAESVRHFALAGRFTDESVAFQTRML : 141
SbKCS9    ------------------ALVFLGTVLRRLLRRQRPVYLLNYSCHLPDAERQVNLEVCEYFGLKCRRYSDDI-DFMRLVY :  90
SbKCS10   --------------MAALLLFNRRFRRRAAAATDDDIGLVDFSCLKPPRRLRLPVAGLLEHLRLIGCFDDGSVEFMAKVI : 109
SbKCS11   AGSLRAV-PPVRILTAVILT--AGVAQLRRLRRPRDVYLVEYGCFRPKPCFRAPFATCLEHAFYLPYQVDPEVTFSIRLL : 123
SbKCS12   LALVREI-RPVHCLLVLLLA--AAVAKLWRMGRPKDVYLVEYGCFRPKPCFRAPFATCLEHAHLMPYLVDEEVSFAIRLL : 118
SbKCS13   -VADLWALRLNLISVVACSTLLVFLGTVYFLTRPRPVYLVDFACYKPGPERRCTRDTFMRCSRLTGCFTDGSLEFQRKIL : 148
SbKCS14   ---VTSAFASNPPFAVSLLVLSAVLATAYLMRRPRAVYLLDFACYKPGAGHVVTRETFMRQSEAAGVFTPDNLAFQRKIL : 145
SbKCS15   DLIVSARLTANLPFTV--VVVAAAATAVYLMRRPRAVYLLDLACYKPGAEHAVTRETFMRLVVGYGTFSDDSLAFQRKIL : 140
SbKCS16   -----------------------------------------------EPRHRVPFATALEHGRLMNDLIDEELSFMVRLH :  33
SbKCS17   SGLL----PIHVLMLMVVLV--AGLAMIWRLHRPRDVYLVDYGCFLGDPCYRVPFAMALEHGRLMTDLIDEELSFMVRLH : 101
SbKCS18   LRQLWLHLQYNLVSVLVLSAVLVFGATVYVLTRPRPVYLVDFACYKPPAHLQVRFEEFMRHSKLCG-FSEDALEFQRKIL : 148
SbKCS19   -LADLWQLQYNLVSVLVCTTVLVVVATAYALTRPRPVYLVDFACYKPDDERKCSRARFMNCTESLGTFTPENIEFQRRII : 140
SbKCS20   AGQFREA-RPVHVFLAAFVP--AAAATVYLMLRPRAVYLVDYACFRTASNCRVPFSTFLEHAKQV-PVLDERIRFMTKLL : 123
SbKCS21   IFPLHAL-RPIYLLLTALLP--AAAFTLYLVNRSHDVYLVDYACFKPNINCRVPLAAFKEHMMHLRPFLDDRVHFLTRMI : 120
SbKCS22   TTLLHSV-SNTDVLSIGLLL--GTAAVVAIMRRPRAVYLIDYACFRPPHTNRVPAAAFVEHVQHV-SQLTERKRFLTRLY : 120
SbKCS23   LIWLHAV-THAHLLLLTFLLSIGLGILVYFMYTSHHVYLVDYACFRHTANCRVSMASFIEHLHHM-PSLNDNIQFMTRMI : 121
SbKCS24   TLSTNNL-PPIHHLLVFLLI--TVVTVKYLIRHPRRVYLVEYTCFGPDSKYRVAPASMIEYFHLA-NLLDDDISFLSNIY :  94
SbKCS25   -LADLWELRFNLLSVVACSTLLVFLSTVYFLTRPRPVYLLDFACYKPEPERKCTRQTFMHCSNLTGSFTDDNLEFQRKIL : 144
```

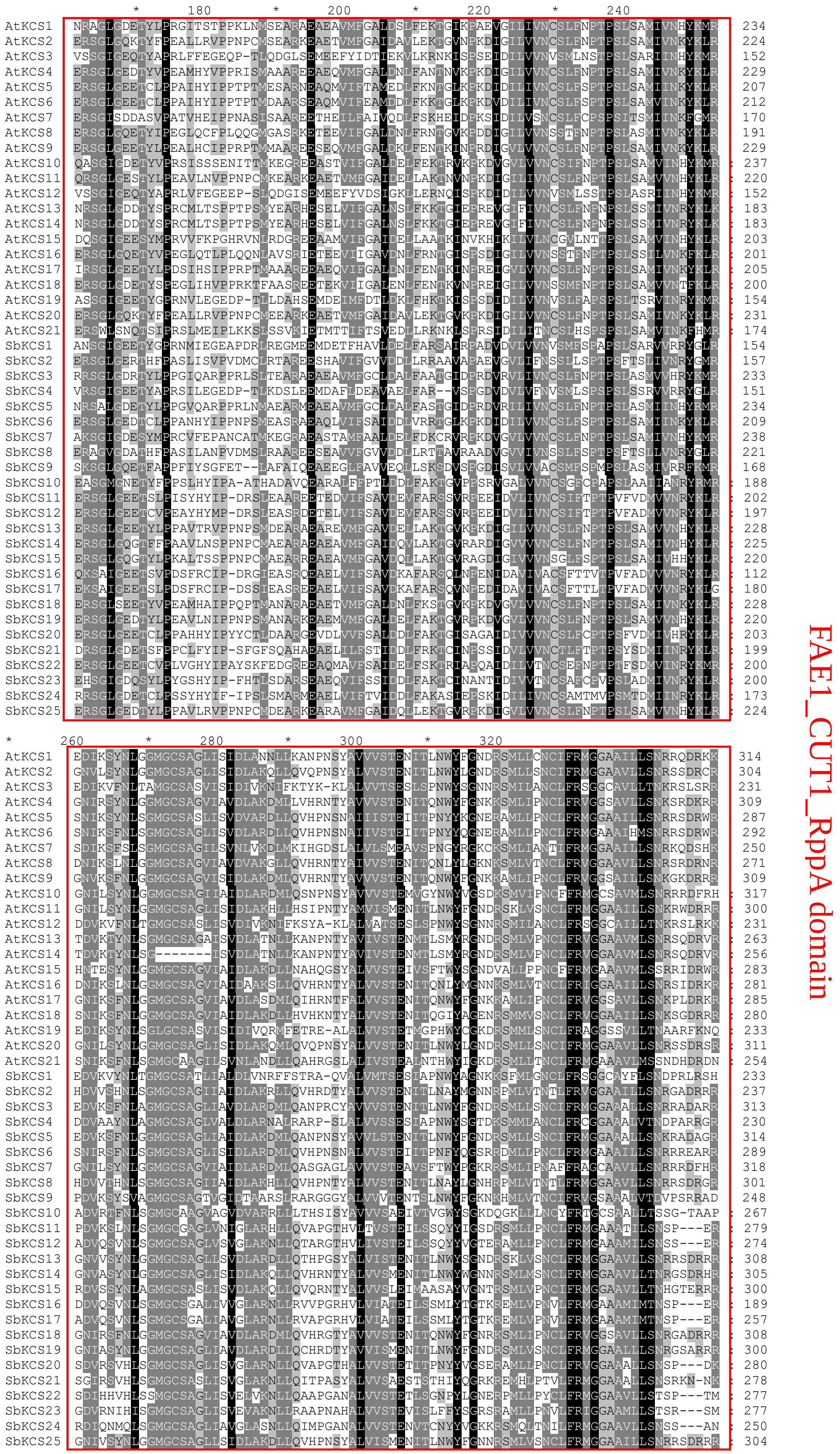
FAE1_CUT1_RppA domain

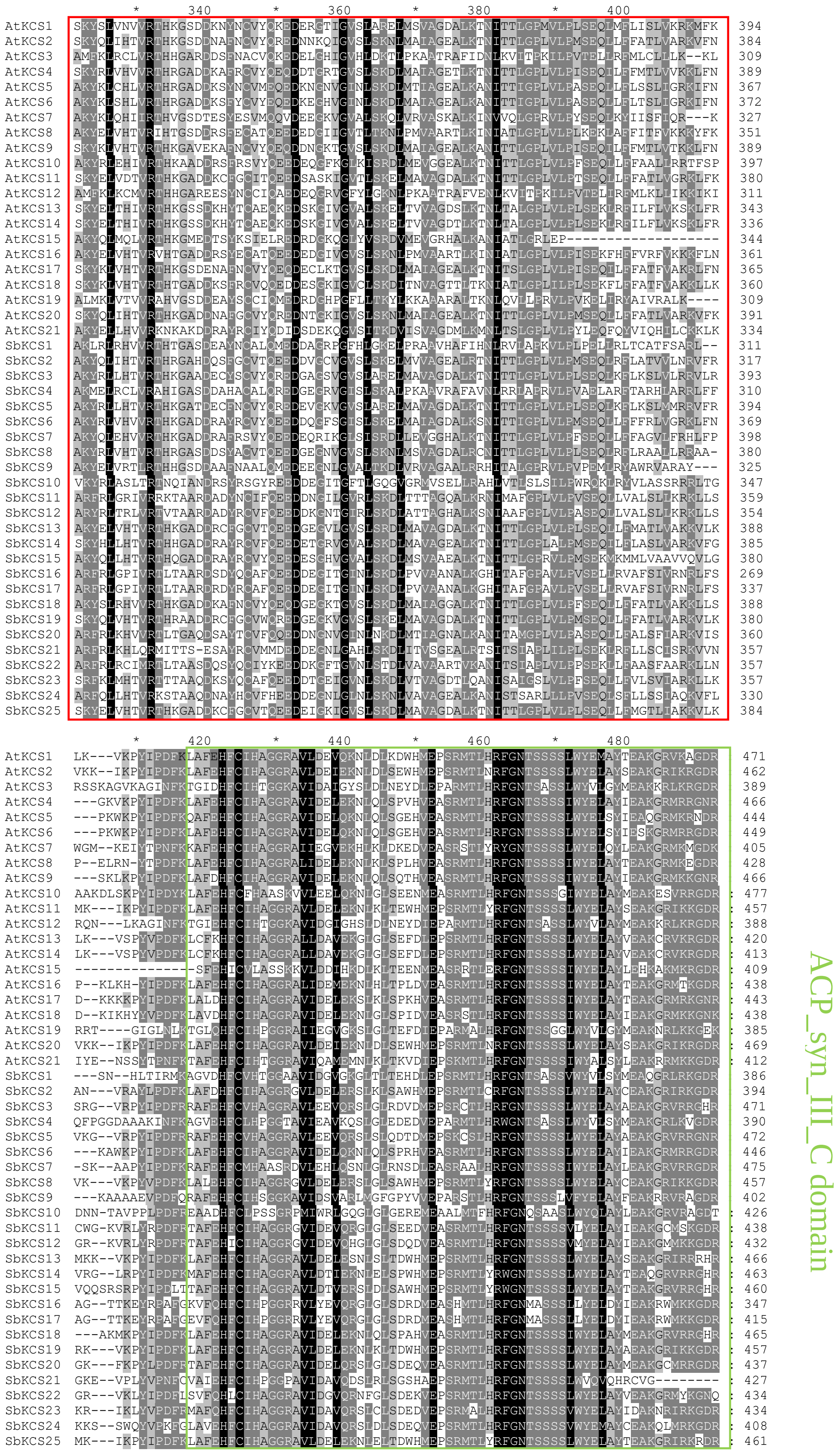

ACP_syn_III_C domain

```
                 *      500        *      520        *
AtKCS1   LWQIAFGSGFKCNSAV[illegible]KALRPVSTEEMTNAWAGSIDQYPVKVVQ----------- : 516
AtKCS2   TWQIAFGSGFKCNSAVWRALRTID-PSKENPWIDEIHEFPVPVPRTSPVTSSSESR : 517
AtKCS3   VFMISFGAGFKCNSCVWEVVRDLNVGEA-NVWNHCINQYPPKS-ILNPFFEKYGWI : 443
AtKCS4   VWQIAFGSGFKCNSAIWEALRHVK-PSNNSPWEDCIDKYPVTLSY----------- : 510
AtKCS5   VWQIAFGSGFKCNSAVWKCNRTIK-TPTDGAWSDCIERYPVFIPEVVKL------- : 492
AtKCS6   VWQIAFGSGFKCNSAVWKCNRTIK-TPKDGPWSDCIDRYPVFIPEVVKL------- : 497
AtKCS7   VWQIGFGSGFKANSAVWKCISEIDSRGR-NAWSDRIHLYPVCGDTSSALKTELLS- : 459
AtKCS8   IWQIALGSGFKCNSSVWVALRDVK-PSANSPWEDCMDRYPVEIDI----------- : 472
AtKCS9   VWQIAFGSGFKCNSAVWVALNNVK-PSVSSPWEHCIDRYPVKLDF----------- : 510
AtKCS10  VWQIAFGSGFKCNSVVWKAMRKVKKPTRNNPWVDCINRYPVPL------------- : 520
AtKCS11  IWQIAFGSGFKCNSSVWRAVRSVN-PKKENPWMDEIHEFPVEVPKVSTI------- : 505
AtKCS12  VFMISFGAGFKCNSCVWEVVRDLTGGES-NVWNHCIDDYPPKS-ILNPYLEKFGWI : 442
AtKCS13  VWQLAFGSGFKCNSIVWRALRTIPANESLNPWGDSVHKYPVHVT------------ : 464
AtKCS14  VWQLAFGSGFKCNSIVWRALRTIPANESLNPWGDSVHKYPVHVT------------ : 457
AtKCS15  VWQIGFGSGFKCNSVVWKALKNIDPPRHNNPWNL---------------------- : 443
AtKCS16  IWQIALGSGFKCNSSVWVALRNVK-PSTNNPWEQCLHKYPVEIDIDLKE------- : 486
AtKCS17  VWQIAFGSGFKCNSAVWVALRNVE-PSVNNPWEHCIHRYPVKIDL----------- : 487
AtKCS18  AWQIALGSGFKCNSAVWVALRNVK-ASANSPWQHCIDRYPVKIDSDLSKSKTHVQN : 493
AtKCS19  ILMMSMGAGFESNNCVWEVLKDLD-----NVWEDSVDRYPELSRIPNPFVEKYDWI : 436
AtKCS20  TWQIAFGSGFKCNSAVWKALRTID-PMDENPWIDEIDDFPVQVPRITPITSS---- : 520
AtKCS21  VLQIAFGSGFKCNSAVWRCIRKVEPNTE-NKWLDFIDSYPVDVPDSTNIRPG---- : 463
SbKCS1   VLMVTFGAGFKCNSCIWTVENPAT-----GVWKDRIHLYPLKD-VSNPFMEKYGFL : 436
SbKCS2   VWQIAFGSGFKCNSAVWKALRTVD--GGENPWTPEMDVLPIHVPKVSPIDETTYTF : 448
SbKCS3   VWQIGFGSGFKCNSAVWRALRDVPPVHADNPWVDSIHRYPPKAYI----------- : 516
SbKCS4   VLMVTFGSGFKCNSCVWEVTGDMA-----GAWADCIDDYPPET-LANPYMDKFGWI : 440
SbKCS5   VWQIGFGSGFKCNSAVWRALQDVPALSSSNPWVDDVDRYPPKAYV----------- : 517
SbKCS6   VWQIGFGSGFKCNSAVWKCLRSIK-TPTNGPWDDCIHRYPVDVPEVVKL------- : 494
SbKCS7   VWQLAFGSGFKCNSAVWRAVRRVRRPAR-SPWLDCVDQYPARMDA----------- : 519
SbKCS8   VWQIAFGSGFKCNSAVWKALRTVDDAARENPWADEIDDLPVHVPKVSPFDSSD-QQ : 512
SbKCS9   LWMLAFGTGFKACSNVWRALRDAAP--D-NPWNGCVHRYPVPP-PPPSKTHKHA-- : 452
SbKCS10  VWQLGIGSGLKANSLVWERVAGHEDEGERGPWKDCIHKYPVKGT------------ : 470
SbKCS11  VWMISFGSGFNCSSVAWECLKPAIDSD--GPWADCIHRYPVQLPDVLDGSIA---- : 488
SbKCS12  IWMISFGAGFDCNSVAWECVKPPADAD--GPWVDSIHRYPVQLPEIVKDTSDDS-- : 484
SbKCS13  VWQIAFGSGFKCNSAVWRALRSVN-PAEENPWMDEIDRFPVDVPKVSKVTSD---- : 517
SbKCS14  AWQIAFGSGFKCNSAVWRALRTID-PKKENPWVEEIHRFPVEVPKVESVIVS---- : 514
SbKCS15  VCQIAFGSGFKCNSAVWRALRTID-PAKENPWVEEIHRFPVEVPQVERVDVAAMAS : 515
SbKCS16  LCMISFSPGIDCSSVVWECVKPTADTDS-GPWAGCIHRYPVQLPKIVERA------ : 396
SbKCS17  LCMISFSPGIDCSSVVWECVKPTADTDS-GPWAGCIHRYPVQLPKIVERA------ : 464
SbKCS18  IWQIAFGSGFKCNSAVWHALRNVK-PSPDNPWEDCIDRYPVELVDGFPTHKPQQ-- : 518
SbKCS19  TWQIAFGSGFKCNSAVWKALRSVN-PAKENPWRDDIDRYPVAVPKVSAI------- : 505
SbKCS20  VWMIGFGSGFKCNSAAWECIEPARTAE--GPWATSIHRYPVHIPDVMKH------- : 484
SbKCS21  ---------------VYPTIPQC--------------------------------- : 435
SbKCS22  VWMIGFGSGFKCNSAVWECIRPVHDAH--GPWANCIDRYPVHIP------------ : 476
SbKCS23  IWMIGFGSGFKCNSVVWKCIKSASNLD--GPWADCIHRYPTK-------------- : 474
SbKCS24  VWMIGFGSGYKCNSAVWKCILPARSAD--SAWANCIHRYPMEVPKQV--------- : 453
SbKCS25  IWQIAFGSGFKCNSAVWKALRTVN-PAKESPWMDEIDNFPVDVPKISKVGGA---- : 512
```
